# Neurological phenotypes in patients with NLRP3-, MEFV-, and TNFRSF1A low-penetrance variants

**DOI:** 10.1186/s12974-020-01867-5

**Published:** 2020-06-20

**Authors:** Elisabeth Mulazzani, Danny Wagner, Joachim Havla, Miriam Schlüter, Ingrid Meinl, Lisa-Ann Gerdes, Tania Kümpfel

**Affiliations:** 1 Institute of Clinical Neuroimmunology, Biomedical Center and University Hospital, Ludwig-Maximilians University, Marchioninistraße 15, 81377 Munich, Germany; 2 Munich Cluster for Systems Neurology (SyNergy), Munich, Germany

**Keywords:** CAPS, TRAPS, FMF, Multiple sclerosis, Autoinflammation, Autoimmunity

## Abstract

**Background:**

Neurological manifestations and the co-occurrence of multiple sclerosis (MS) have been reported in patients with autoinflammatory diseases (AID) and variants of the *NLRP3*-, *MEFV*-, or *TNFRSF1A* gene. However, type and frequency of neurological involvement are widely undetermined.

**Methods:**

We assessed clinical characteristics of 151 (108 with MS) patients carrying NLRP3-, MEFV- and TNFRSF1A low-penetrance variants  from the Institute of Clinical Neuroimmunology. We evaluated demographic, genetic, and clinical features with a focus on central nervous system (CNS) involvement including magnetic resonance imaging (MRI) results and cerebrospinal fluid (CSF) data. The disease course of AID patients with MS was compared to a matched MS control group without mutations.

**Results:**

The genetic distribution comprised 36 patients (23%) with NLRP3- and 66 patients (43%) with TNFRSF1A low-penetrance variants as well as 53 (34%) patients carrying pathogenic mutations or low-penetrance variants in the *MEFV* gene. MS patients displayed most frequently the R92Q TNFRSF1A variant (*n* = 51; 46%) followed by the Q703K NLRP3 variant (*n* = 15; 14%) and the E148Q substitution (*n* = 9; 8%) in the MEFV gene. The disease course of MS was not influenced by the genetic variants and did not differ from MS patients (*n* = 51) without mutations. AID patients without MS most frequently harbored MEFV mutations (*n* = 19, 43%) followed by NLRP3- (*n* = 17, 39%) and TNFRSF1A (*n* = 8, 18%) low-penetrance variants. Sixteen (36%) of them suffered from severe CNS involvement predominantly recurrent aseptic meningoencephalitis and optic neuritis accompanied by abnormal MRI and CSF results. Severe CNS inflammation was associated with the Q703K allele. Headache was a highly prevalent neurological symptom (up to 74%), irrespective of the underlying genetic variation. The NLRP3 cohort without MS more frequently exhibited affections of the cranial nerves (CN) (*p* = *0*.*0228*) and motor symptoms (*p* = *0*.*0455*). Elevated acute-phase reactants were detected in all patients, and fever episodes were present in up to 50%. Arthralgias were the most frequently identified constitutional symptom among all subgroups.

**Conclusions:**

Our data highlight the high prevalence of neurological manifestations, including concomitant MS, among NLRP3-, MEFV-, and TNFRSF1A low-penetrance variants. In particular, patients carrying the Q703K NLRP3 variant are at risk for severe CNS inflammation and CN affection.

## Background

Cryopyrin-associated periodic syndromes (CAPS) including Muckle-Wells syndrome (MWS), familial cold autoinflammatory syndrome (FCAS) and neonatal-onset multisystem inflammatory disease (NOMID/CINCA), familial Mediterranean fever (FMF), and tumor necrosis factor receptor-associated periodic syndrome (TRAPS), all belong to a group of rare, monogenetic autoinflammatory diseases (AID) caused by a dysregulation of the innate immune system. While CAPS is mediated via gain-of-function mutations in the *NLRP3* gene leading to a constitutively activated NLRP3 inflammasome, FMF is caused by sequence variants in the *MEFV* locus encoding the inflammasome complex pyrin [1, 2]. At the molecular level, NLRP3 serves as an intracellular danger signaling complex, while pyrin acts as a sensor detecting an imbalance in RhoGTPases activity [3]. Despite this diverging signaling pathway upstream of inflammasome activation exists, both CAPS and FMF result in a common overproduction of interleukin 1β (IL-1β) [4, 5]. On the contrary, TRAPS is caused by mutations in the *TNFRSF1A* gene, which encodes the tumor necrosis factor receptor (TNF) receptor, and has formerly been associated with an accumulation of mutated TNFR1 receptors in the endoplasmic reticulum due to inadequate shedding, thus triggering inflammatory response [6–8].

Clinically, AID are characterized by unexplained episodes of fever and systemic inflammation involving joints, skin, muscles, eyes, and serosal surfaces accompanied by elevated acute-phase reactants [9]. Neurological manifestations with the involvement of the central nervous system (CNS) including aseptic meningitis, headache, increased intracranial pressure, seizures, cerebral vasculitis, and inflammatory lesions in the brain have mainly been reported in CAPS, but are also present in FMF and TRAPS and may even dominate the clinical picture [10–18]. Furthermore, cases with multiple sclerosis (MS)-like presentations have been observed for all three of them [17, 19–21]. The importance of variants with unknown pathogenic significance (such as p.R92Q in *TNFRSF1A* or p.Q703K in *NLRP3*) is still under debate, and their clinical phenotypes are often challenging for clinicians [22, 23]. In recent studies, we could describe several patients with NLRP3-, MEFV-, and TNFRSF1A low-penetrance variants in association with inflammatory diseases of the CNS including MS [24–29].

The aim of this study was to evaluate genotype-phenotype correlations focusing on neurological manifestations in a monocentric cohort of 151 patients (with and without (w/o) MS) with predominantly low-penetrance mutations in the *NLRP3*-, *MEFV*-, or *TNFRSF1* gene.

## Methods

All patients were consecutively seen and examined by an experienced neurologist at our neuroimmunological outpatient clinic from 2006 to 2020, which is specialized on the diagnosis and treatment of adult patients with MS and other neuroimmunological diseases. Inclusion criteria encompassed clinical presentation suggestive of AID (≥ 2 symptoms compatible with AID) and the genetic proof of a variant in the *NLRP3*-, *MEFV*-, or *TNFRSF1A* genes. Genetic testing included sequencing of exons 3, 4, and 6 of the *NLRP3* gene as well as exons 2, 3, and 10 of the *MEFV* gene and exons 2, 3, 4, and 6 of the *TNFRSF1A* gene. Patients were then grouped according to their AID and MS status into NLRP3-, MEFV-, and TNFRSF1A variants with and w/o MS. Patients harboring genetic mutations in two different genes were accordingly allocated to both subgroups. In addition, MS disease course of mutation carriers was compared to a matched MS control group (*n* = 51) lacking genetic variants in the *NLRP3*-, *MEFV*-, and *TNFRSF1A* genes.

Demographic data, family history, clinical characteristics, MRI, and laboratory findings (cerebrospinal fluid [CSF] and blood), as well as treatment information, were collected during standardized visits at our institute. In patients with MS, data on the disease course were evaluated using the Expanded Disability Status Scale (EDSS) and the Multiple Sclerosis Severity Scale (MSSS) was evaluated [30]. MRI and CSF data were analyzed in more detail in AID patients w/o MS, and CNS involvement was classified as mild (only clinical signs/symptoms, normal CSF, no MRI abnormalities) or severe (clinical signs/symptoms, abnormal CSF and/or MRI). In addition, neurological symptoms were specifically grouped into 5 categories: sensory and motor symptoms, cerebellar signs, cranial nerve (CN) affection, and headache. The term CN affection itself was defined as specific inflammation of the cranial nerves I-XII in patients with AID and CNS manifestation w/o MS. Clinical characteristics of constitutional symptoms were also assessed in all patients including disease course (chronic vs. episodic), fever, skin involvement, abdominal pain, arthralgias, myalgias, and ocular involvement (conjunctivitis/uveitis/retinitis/papillitis). All patients were classified according to the most recent classification criteria for AID [23]. Patients were followed over time whenever possible. Follow-up time of AID patients with MS (mean 10.5 ± 4.6) and of MS control group (mean 9.2 ± 2.8) ranged from 2 to 18 years. Mean follow-up time of AID patients w/o MS was 8.7 ± 3.1 (4–15) and exact follow-up time for each patient is shown in suppl. fig. 1.

Frequencies and percentages were used as descriptive statistics for categorical variables. Median and range were used to describe numerical variables. In order to analyze the clinical phenotype of the patients in relation to their gene variants and MS disease status, we divided the patients into six subgroups: NLRP3 variants without (w/o) or with MS, MEFV variants w/o or with MS, TNFRSF1A w/o or with MS. Intergroup comparisons of main clinical characteristics were assessed by Fisher’s exact test or chi^2^ test, as appropriate. All tests were two-sided. All analyses were performed with Prism Software (GraphPad©). The Benjamini-Hochberg procedure was used to correct for multiple comparisons, and the threshold for statistical significance was set to *p* < 0.05.

## Results

### Demographic and general clinical characteristics

Altogether, 151 patients (40 males, 111 females mean age 44, range 20–75 years) with low-penetrance variants or pathogenic mutations in the *NLRP3*-, *MEFV*-, or *TNFRSF1A* gene and clinical presentation suggestive for AID were included in the study. One hundred and eight patients had a concomitant diagnosis of MS. Four of 151 patients carried a double mutation (1 patient w/o MS: *NLRP3* and *TNFRSF1A* variants, 3 patients with MS: two with MEFV and TNFRSF1A variants, one with MEFV and NLRP3 variants) leading to 155 AID in total (AID w/o MS = 44; AID with MS = 111) (Table 1). The majority of patients were adults with a median age at AID-related symptom onset of 27.5 ± 6.8 years consistent with late-onset disease manifestation. None of the patients were diagnosed before the age of 6 months. The longest diagnostic latency for AID was observed in MS patients with MEFV variants with a mean of 12.3 ± 11.4 years. Only 38 (25%) patients were of Mediterranean origin, most of them carried MEFV variants (*n* = 22). Overall family history for AID was positive in 68 (44%) patients, with more affected family members in patients with NLRP3 variants w/o MS (65%, see Table 1). A total of 17 (11%) patients (4 with MS) were treated with anti-IL1 drugs (anakinra or canakinumab), while 20 (13%) patients (all with MEFV variants, 4 with MS) received colchicine. Detailed characteristics of all patient groups are presented in Table 1.

**Table 1:** Study population

Features	All variants (***n*** = 155)	NLRP3 variants w/o MS (***n*** = 17, 11%)	NLRP3 variants with MS (***n*** = 19, 12%)	MEFV variants w/o MS (***n*** = 19, 12%)	MEFV variants with MS (***n*** = 34, 22%)	TNFRSF1A variants w/o MS (***n*** = 8, 5%)	TNFRSF1A variants with MS (***n*** = 58, 38%)
M:F	1:2.8	1:4.6	1:1.7	16:5.3	1:3.4	All females	1:1.4
Age at AID onset (years)	27.5 ± 6.8 (2–70)	38.3 ± 16 (12–70)	30.9 ± 13.6 (12–70)	18.7 ± 9.7 (2–33)	22.8 ± 12.3 (6–46)	28.7 ± 12.9 (14–48)	25.9 ± 11.1 (7–55)
Age at AID diagnosis (years)	37.7 ± 5.2 (20–76)	46.1 ± 12.9 (24–71)	40.6 ± 10.8 (20–61)	31.3 ± 10.7 (20–59)	34.3 ± 12.6 (13–63)	36.8 ± 11.6 (16–51)	36.8 ± 14.2 (14–76)
Diagnose latency for AID (years)	10.1 ± 1.8 (1–26)	7.2 ± 7.3 (1–22)	11.1 ± 12.1 (1–36)	10.8 ± 10.3 (0–29)	12.3 ± 11.4 (0–36)	8.7 ± 11.9 (0–28)	10.4 ± 9.3 (0–34)
Disease duration (years)	16.5 ± 3.1 (2–45)	11.1 ± 8.3 (2–27)	14.6 ± 12.7 (2–39)	17.4 ± 12 (3–41)	19.1 ± 10.9 (6–40)	17 ± 10.4 (6–34)	19.5 ± 10.5 (4–45)
Positive family history for AID	68/44%	11/65%	8/42%	10/53%	13/38%	3/38%	23/40%
Mediterranean origin	38/25%	3/18%	4/21%	12/63%	10/29%	0	9/16%
Anti-IL-1 treatment	17/11%	10/59%	2/11%	1/5%	2/6%	3/38%	0
Colchicine	20/13%	0	0	16/84%	4/12%	0	0

Demographic data of 151 patients were assessed. Patients were categorized due to their (1) underlying mutation and (2) MS status as follows: (a) variant in the *NLRP3* gene±MS, (b) variant or mutation in the *MEFV* gene±MS, and (c) variant in the *TNFRSF1A* gene±MS. Numbers (despite M to F ratio) represent mean ± SD (range). Four patients of 151 carried a double mutation (1 patient w/o MS: *NLRP3* and *TNFRSF1A* variants; 3 patients with MS: two with MEFV and TNFRSF1A variants, one a MEFV and NLRP3 variant) and were accordingly counted in both columns/groups (*n* = 155)

### Genetic data

In total, 4 different variants were found in the *NLRP3* gene, 15 mutations were identified in the *MEFV* gene, and 6 genetic variants were found in the *TNFRSF1A* gene. The vast majority of patients carried low-penetrance mutations and was heterozygous for the respective mutation.

**Fig. 1:**
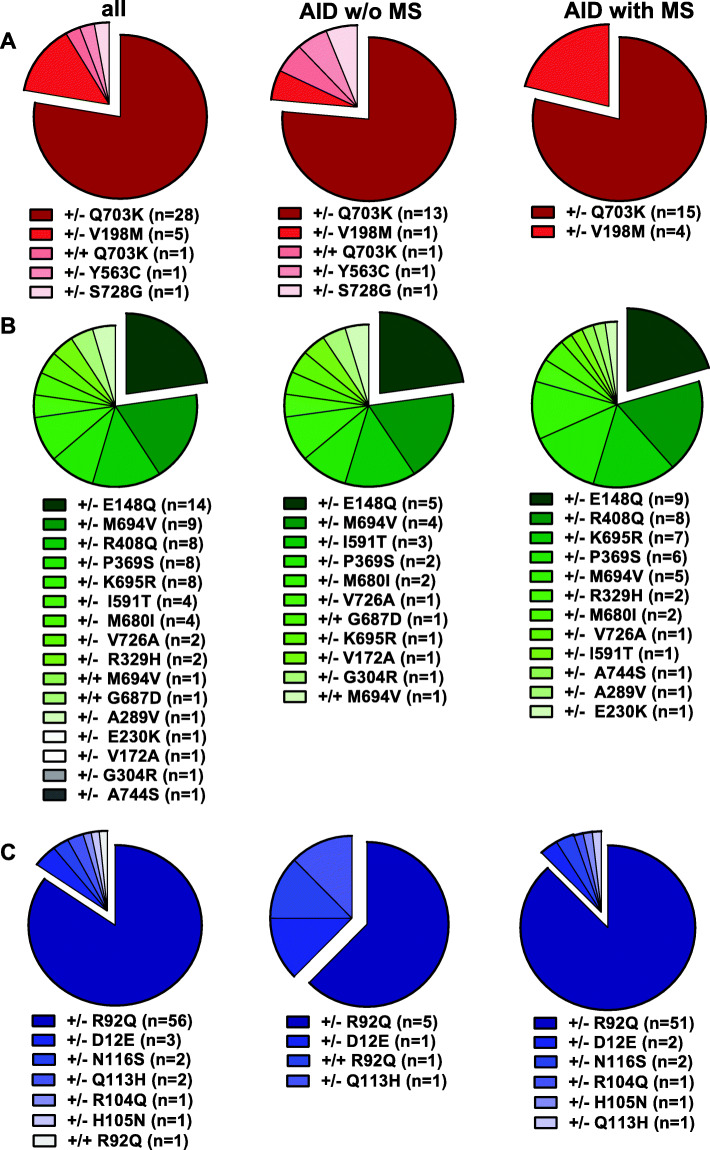
Frequencies of NLRP3-, MEFV-, and TNFRSF1 low-penetrance variants. All patients had sequence variants in *NLRP3* (exon 3, 4, 6), *MEFV* (exon 2, 3, 10), and/or *TNFRSF1A* (exon 2, 3, 4, 6) gene and were categorized due to their underlying mutation and their MS status (AID w/o or with MS). Pie charts demonstrate absolute numbers of mutations. **a** Red slices represent NLRP3 variants. **b** Green slices show different MEFV variants, while **c** TNFRSF1A variants are symbolized by blue charts. Homozygous variants are marked as +/+, heterozygous variants are labeled by +/− symbols

In detail, 36 patients (23%) showed a low-penetrance variant in the *NLRP3* gene (78% carried a Q703K variant in exon 3), 53 patients (34%) exhibited pathogenic mutations or low-penetrance variants in the *MEFV* gene (26% with the E148Q variant in exon 2) and 66 patients (43%) showed variants in the *TNFRSF1A* gene (85% with the R92Q variant in exon 4). A complete overview of all genetic variants is given in Fig. 1a-c. In 13 (25%) FMF patients (10 w/o MS, three with MS), a compound mutation was identified. Severe disease-causing FMF mutations including M694V (+/–), M694V (+/+), K695R (+/-), and M680I (+/–) were found in 22 (42%) MEFV mutation carriers.

AID patients w/o MS (*n* = 40) most frequently harbored MEFV mutations (*n* = 19, 43%) followed by NLRP3- (*n* = 17, 39%) and TNFRSF1A (*n* = 8, 18%) low-penetrance variants (Fig. 1). AID patients with MS (*n* = 111) displayed most frequently the R92Q variant (*n* = 51; 46%) in the *TNFRSF1A* gene followed by the Q703K variant in the *NLRP3*- (*n* = 15; 14%) and the E148Q variant (*n* = 9; 8%) in the *MEFV* gene (Fig. 1). The MEFV low-penetrance variants K695R (*n* = 7) and R408Q (*n* = 8, in six cases as a compound heterozygous P369S-R408Q variant) were almost exclusively found in the MS cohort (Fig. 1b).

### Neurological manifestation

#### Patients without MS

Taken together, 95% of AID patients w/o MS showed neurological manifestations, 16 (36%) of those suffered from severe CNS involvement. Genetically, significantly more patients with severe CNS involvement harbored a Q703K variant in the *NLRP3* gene (*n* = 11; 69%) compared to MEFV- (*n* = 3, 19%) and TNFRSF1A (*n* = 2, 13%) low-penetrance variants (chi^2^ test: *p* = 0.0228). The clinical picture of severe CNS involvement was dominated by optic neuritis (*n* = 11; 69%) and aseptic meningitis/meningoencephalitis (*n* = 5; 31%) while one patient suffered from cerebral vasculitis (*n* = 1; 3%).

**Fig. 2:**
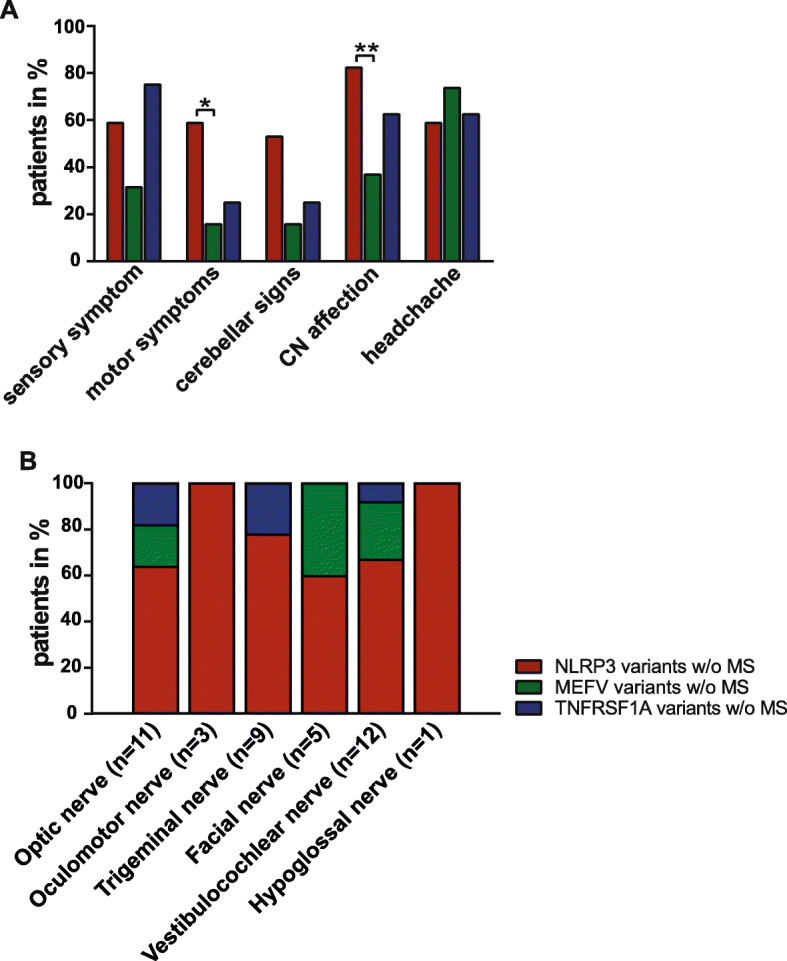
Neurological symptoms of AID patients w/o MS. Neurological symptoms were distinguished as follows: **a** sensory and motor symptoms, cerebellar signs, cranial nerve (CN) affection and headache syndromes in NLRP3-, MEFV-, and TNFRSF1A low-penetrance variants w/o MS. **b** Distribution of patients w/o MS and NLRP3-, MEFV-, and TNFRSF1A low-penetrance variants and CN affection are shown as percentages

Headache syndromes were frequently observed among all AID subgroups w/o MS and most commonly found in patients with MEFV- (*n* = 14; 74%) followed by TNFRSF1A- (*n* = 5; 63%) and NLRP3 low-penetrance variants (*n* = 10, 59%). Patients with NLRP3 variants were also more commonly affected by motor symptoms (NLRP3 variants: *n* = 10, 59%; vs. MEFV variants: *n* = 3, 16%; *p* = 0.0455) compared to MEFV low-penetrance variants (Fig. 2a). In NLRP3 low variant carriers, CN affection was significantly more often identified compared to MEFV patients (NLRP3 variants: *n* = 14, 82%; vs. MEFV variants: *n* = 7, 37%; *p* = 0.0228). Distribution of NLRP3-, MEFV-, and TNFRSF1A low-penetrance variant carriers regarding CN affection are shown in Fig. 2b.

**Fig. 3:**
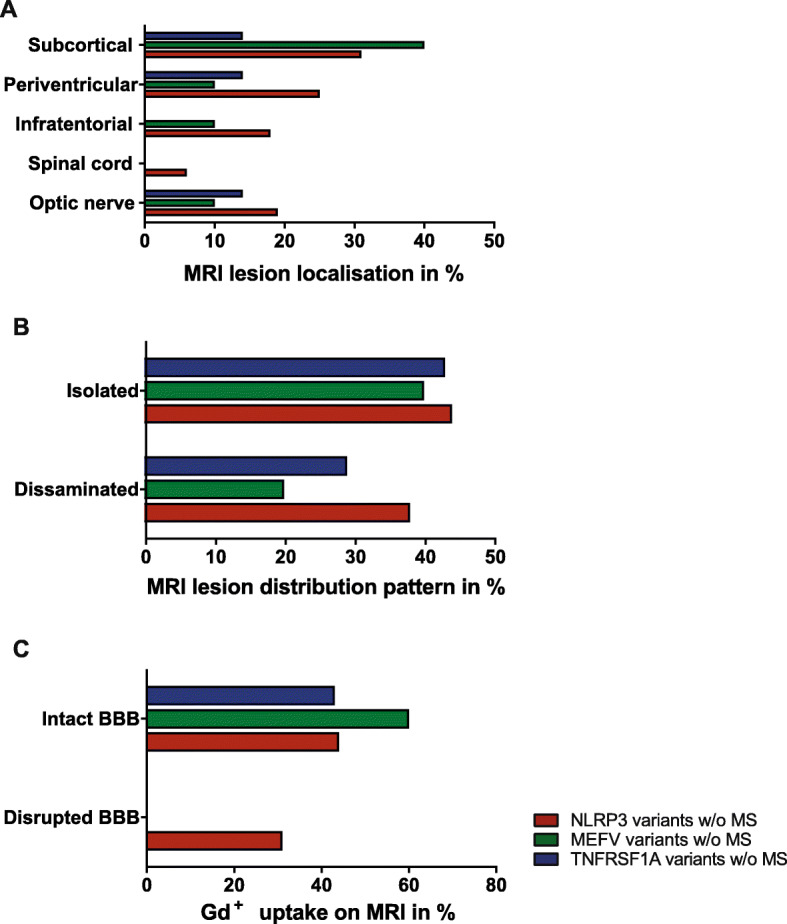
MRI data of AID patients w/o MS. MRI data of low-penetrance mutation carriers in the NLRP3-, MEFV-, or TNFRSF1A gene w/o MS are shown as percentages

MRI data were available for 33 (75%) AID patients w/o MS (NLRP3 variants: *n* = 16; 94%, MEFV variants: *n* = 10; 53%, and TNFRSF1A variants: *n* = 7; 88%). Abnormal MRI findings were present in 11 (69%) patients with NLRP3 low-penetrance variants, in six (60%) patients with MEFV variants, and in three (43%) patients with TNFRSF1A variants. Unspecific white matter lesions were the most common neuroradiological feature. A disrupted blood-brain barrier indicated by an increased gadolinium uptake was only seen in patients with the Q703K NLRP3 variant. Further morphological MRI features are listed in Fig. 3.

**Fig. 4:**
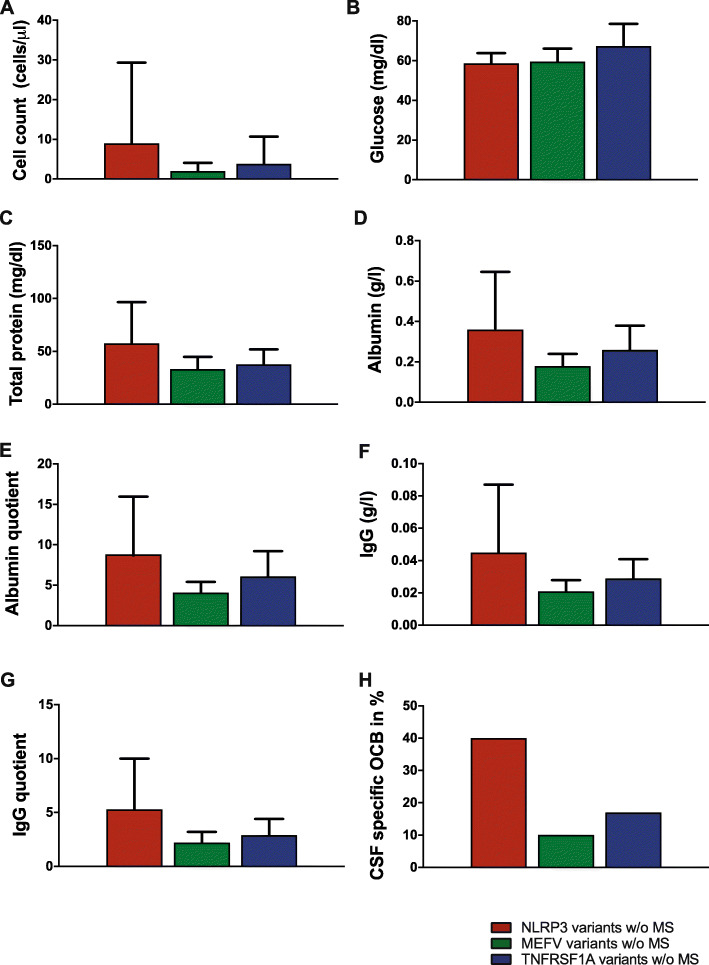
CSF data of AID patients w/o MS. CSF data of low-penetrance mutation carriers in the NLRP3-, MEFV-, or TNFRSF1A gene w/o MS are shown as mean ± SD or as percentages (OCB)

CSF data were available in 31 (71%) AID patients w/o MS (NLRP3 variants: *n* = 15; 88%, MEFV variants: *n* = 10; 53%, and TNFRSF1A variants: *n* = 6; 75%). Pathological CSF findings were present in ten of NLRP3- (67%), two of MEFV- (20%), and two of TNFRSF1A (33%) low-penetrance variants (Fig. 4).

#### MS patients

**Fig. 5:**
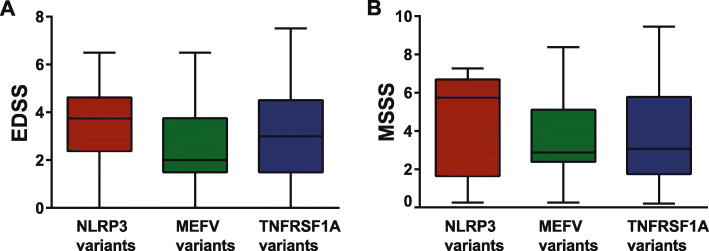
Disability and severity scores of AID patients with MS. Expanded Disability Status Scale (EDSS) and the Multiple Sclerosis Severity Scale (MSSS) in NLRP3-, MEFV-, and TNFRSF1A low-penetrance variants with MS are shown at initial presentation

A total of 108 patients with AID had a concomitant MS. The majority of patients (*n* = 92) were classified as relapsing-remitting MS (RRMS), twelve had secondary progressive MS (SPMS), and seven patients were diagnosed with primary progressive MS (PPMS). Neurological presentations including headache, as well as MS-related disease course, disability, and severity, scores did not differ among AID groups (Fig. 5a, b) and were comparable to MS patients without mutations (Table 2). MS-related medications consisted of disease-modifying therapies (DMT) for mild disease course in 67 MS patients (60%) and for active disease course in 31 MS patients (28%). A slightly higher proportion of MS patients with AID was more often treated with DMT for active disease compared to the MS control group and also showed more often a positive family history for MS. Detailed clinical features of MS patients with AID and of MS control group are summarized in Table 2.

**Table 2:** Clinical data of NLRP3-, MEFV-, and TNFRSF1A low-penetrance variants with MS

Features	Total AIDs with MS (***n*** = 111)	NLRP3 variants with MS (***n*** = 19)	MEFV variants with MS (***n*** = 34)	TNFRSF1A variants with MS (***n*** = 58)	MS w/o mutations (***n*** = 51)
M:F	1:2.2	1:1.7	1:2.4	1:3.4	1:2
Age at MS diagnosis (years)	29.6 ± 12.3	31.5 ± 11.5 (11–48)	28.4 ± 12.3 (7–63)	28.9.0 ± 12.4 (12–51)	32.7.0 ± 11.7 (14–56)
Diagnose latency for MS (years)	3.3 ± 5.8	4.3 ± 6.7 (0–29)	2.9 ± 5.2 (0–25)	2.8.0 ± 4.0 (0–29)	2.3.0 ± 3.0 (0–29)
MS disease duration (years)	14.6 ± 8.6	12.2 ± 8.6 (3–35)	13.8 ± 8.5 (3–27)	17.7 ± 8.8 (5–32)	14.0 ± 7.8 (8–43)
RRMS	92/83%	18/95%	30/88%	44/76%	39/76%
SPMS	12/11%	1/5%	2/6%	9/16%	7/14%
PPMS	7/6%	0/0%	2/6%	5/8%	5/10%
EDSS at baseline	3 ± 2	4 ± 2	3 ± 2	3 ± 2	3 ± 2
EDSS at follow-up	3 ± 2	3.5 ± 2	3.5 ± 2	3 ± 2	3.3 ± 2
MSSS at baseline	3.1 ± 1.9	4.4 ± 2.6	3.8 ± 2.3	3.8 ± 2.6	3.8 ± 1.8
MSSS at follow-up	3.1 ± 1.9	3.2 ± 2.2	3.4 ± 1.7	2.8 ± 2	2.9 ± 1.5
DMT for mild disease course*	67/60%	10/53%	28/82%	29/50%	35/68%
DMT for moderate/severe disease course**	31/28%	3/16%	6/18%	22/38%	9/18%
No DMT	13/12%	6/32%	0/0%	7/12%	7/14%
Positive family history for MS	23/21%	5/26%	6/18%	12/21%	6/12%

Demographic data of 108 MS patients were assessed. Numbers, if not stated differently, represent mean ± SD (range). *EDSS* Expanded Disability Status Scale, *MSSS* Multiple Sclerosis Severity Score, *RRMS* relapsing-remitting MS, *SPMS* secondary progressive MS, *PPMS* primary progressive MS; *DMT* disease-modifying therapies

*Interferons, glatirameracetat, teriflunomide, dimethylfumarate, steroids, and azathioprine

**Fingolimod, natalizumab, alemtuzumab, ocrelizumab, and rituximab

### Systemic symptoms

**Fig. 6:**
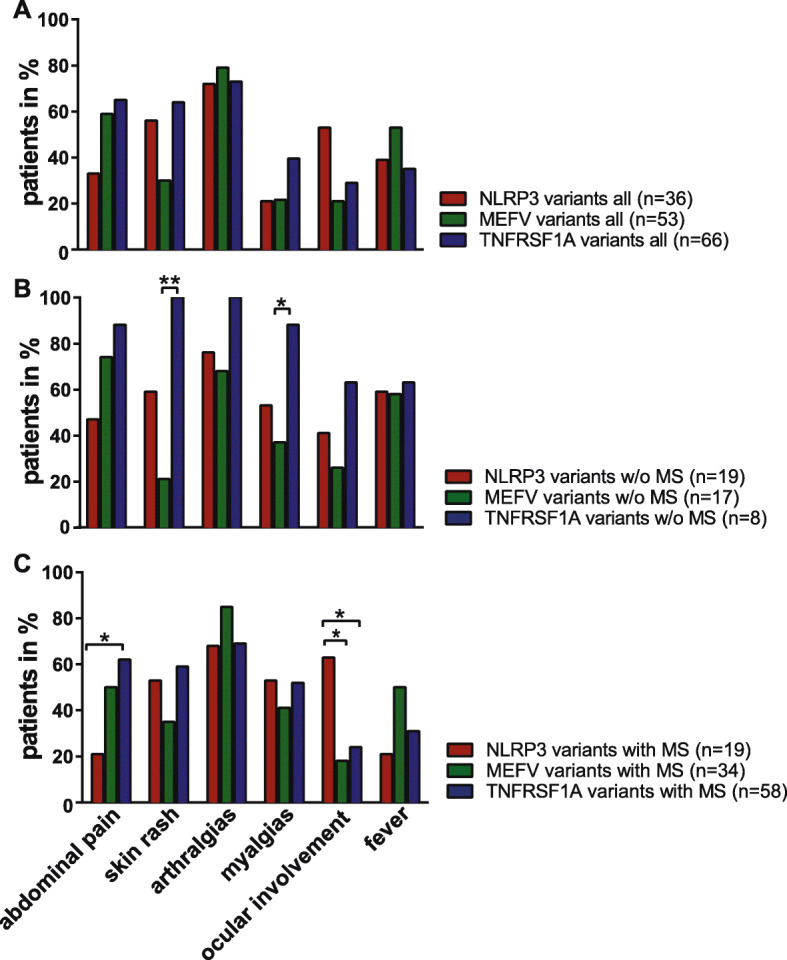
Systemic symptoms. Systemic symptoms were distinguished as follows: abdominal pain, skin rash, arthralgias, myalgias, ocular involvement, fever in all patients (**a**) of NLRP3-, MEFV-, and TNFRSF1 low-penetrance variants w/o (**b**) or with MS (**c**). Numbers represent percentages

Overall 79% (*n* = 119) of patients fulfilled the most recent classification criteria for the respective AID [23]: 58% (*n* = 21) with NLRP3 variants, 60% (*n* = 32) with MEFV low-penetrance or pathogenic mutations, and 100% (*n* = 66) with TNFRSF1A variants. Most patients suffered from an episodic disease course (*n* = 137; 88%) with intermittent flares. Looking at all patients, arthralgias was the most common symptom followed by abdominal pain, myalgias, skin rash, fever and ocular involvement (Fig. 6a). Elevated acute-phase reactants (CRP 2.7 ± 1.6 [range 0.6–86] and SAA 31 ± 36.2 [range 5.2–3115]) were detected in all subgroups (Table 3). None of the patients so far showed evidence of amyloidosis or kidney disease. Twenty-two patients (14%) were also diagnosed with other autoimmune diseases including autoimmune thyroid disease, diabetes mellitus type I, Crohn’s disease and alopecia areata. Four patients had a concomitant diagnosis of a rheumatic disease (two patients with D12E and N116S variants in the *TNFRSF1A* gene had rheumatoid arthritis/polyarthritis, one patient with a M694V MEFV variant was diagnosed with systemic lupus erythematosus, and one patient with a compound P369/R408Q MEFV variant was diagnosed with ankylosing spondylitis).

**Table 3:** Laboratory data NLRP3-, MEFV-, and TNFRSF1A low-penetrance variants

	All	NLRP3 variants w/o MS	NLRP3 variants with MS	MEFV variants w/o MS	MEFV variants with MS	TNFRSF1A variants w/o MS	TNFRSF1A variants with MS
**CRP (mg/dl)**	2.7 ± 1.6 (54) (0.6-86)	2.2 ± 2.0 (7) (0.6-10.1)	2.1 ± 1.2 (3) (0.8-2.8)	4.7 ± 8.9 (10) (0.65-86)	1.7 ± 1.5 (16) (0.6-11.5)	0.7 ± 0.1 (2) (0.7-0.8)	4.7 ± 6.6 (16) (0.6-23.8)
**Leucocytes (G/l)**	13.3 ± 0.6 (36) (10.5–31.9)	13.5 ± 2.4 (6) (10.3–29)	14.1 ± 3.1 (4) (10.7–31.9)	13.2 ± 1.5 (7) (11.1–19.3)	12.5 ± 2.1 (8) (10.5–16.6)	-	13.0 ± 2.1 (11) (11.3-18.6)
**SAA (mg/dl)**	31 ± 36.2 (68) (5.2-3115)	172.2 ± 492.9 (11) (5.2-3115)	21.7 ± 32.8 (8) (5.2-107)	10.5 ± 5.1 (9) (6.0-22.1)	95 ± 298.2 (14) (5.2-1130)	9.4 ± 3.0 (5) (5.7-13-1)	18.4 ± 25 (21) (5.2-122)

Numbers represent mean ± SD (sample size) and (range). *CRP* C-reactive protein (normal up to 0.5 mg/dl); leucocytes (normal up to 10.4G/l); *SAA* serum amyloid-a (normal up to 5.0 mg/l)

#### Patients without MS

Patients w/o MS and with TNFRSF1A variants showed significant more often myalgias (*n* = 7; 88%) compared to patients with MEFV variants (*n* = 7; 37%, *p* = 0.0169). Patients with TNFRSF1A variants (*n* = 8; 100%) also showed significant more often skin rashes compared to patients with MEFV variants (*n* = 4; 21%, *p* = 0.0074) (Fig. 6b).

#### Patients with MS

MS patients with NLRP3 low-penetrance variants (*n* = 4; 21%) suffered less frequently from gastrointestinal symptoms compared to MS patients with TNFRSF1A variants (*n* = 36; 62%, *p* = 0.0169). Ocular involvement specifically uveitis was most commonly identified in NLRP3 low-penetrance variants (*n* = 12; 63%) in comparison to TNFRSF1A- (*n* = 14; 24%, *p* = 0.0169) and MEFV variants (*n* = 6; 18%, *p* = 0.0141) (Fig. 6c). Neither treatment with colchicine nor with anti-IL-1 therapy (Table 1) had a negative impact on the disease course of MS.

## Discussion

This large, monocentric case series demonstrates a broad spectrum and high prevalence of neurological presentations in adult patients with AID predominantly caused by a variety of  low-penetrance mutations in the *NLRP3*-, *MEFV*-, or *TNFRSF1A* gene. Here, 95% of AID patients w/o MS suffered from neurological manifestations. This highlights the importance of neurological assessments in patients with AID and conversely, that neurologists may consider AID in patients with unexplained CNS as well as systemic symptoms caused by inflammation.

Low-penetrance variants occur at a high allelic frequency in the common population [26, 28, 31, 32]. Clinically, they usually present with a “late-onset” of AID and a more heterogeneous spectrum of symptoms, often accompanied by a milder disease course compared to classical pathogenic mutations in patients with AID [33–36]. Our results also show that the majority of patients had late onset of AID and a broad phenotype overlapping among the different subgroups. Nevertheless, one third of our patients had a severe phenotype involving the CNS. There is mounting evidence, based on both experimental and clinical studies, that also non-confirmatory genotypes can exert pro-inflammatory effects and may lead to severe organ involvement including CNS manifestations in a subset of those patients [24, 37, 38]. We observed severe CNS manifestations including recurrent aseptic meningitis/meningoencephalitis and optic neuritis as well as cerebral vasculitis in several patients w/o MS, most of them harboring the Q703K NLRP3 variant. This was underscored by a high number of abnormal MRI and CSF findings in those patients. CNS inflammation is often observed in patients with pathogenic NLRP3 mutations but has also been reported in patients with MEFV mutations [5, 12, 14, 15, 39, 40]. Regarding NLRP3, neurological involvement was so far reported for 40–95% of CAPS patients and has mainly been linked to the pathogenic R260W mutation of the *NLRP3* gene, while the V198M variant was negatively associated with neurological involvement [12, 13, 35]. Thus, the high occurrence of severe CNS inflammation in patients with the low-penetrance Q703K variant compared to patients with other genotypes in our study is novel and unusual. In all our patients, a thorough and intensive diagnostic work-up was performed and other potential differential diagnoses such as neurosarcoidosis or rheumatological diseases such as lupus erythematodes with CNS involvement were ruled out. However, we cannot exclude other genetic factors contributing to the severe phenotype as we neither performed whole-exome sequencing and nor excluded somatic mosaicism in our patients [41].

Looking in more detail on neurological manifestations in patients w/o MS, headache was the most prevalent symptom among low-penetrance variant carriers irrespective of the underlying genotype. This finding demonstrates a similar result compared to AID patients carrying disease-causing variants, of whom headache syndromes were also reported at a high frequency such as 84% of CAPS and 72% of FMF patients [12, 39]. A greater proportion of patients with NLRP3 variants showed CN affection, which supports findings of our previous study and underscores the vulnerability of cranial nerves to inflammation caused by NLRP3-associated inflammasome activation [24]. Studies in animal models have shown that sensory neurons, including the trigeminal nucleus and ganglion, express NLRP3 which upon activation promotes IL-1 release and is blocked by MCC950, a specific NLRP3 inhibitor [42, 43]. Additional studies have also demonstrated that NLRP3 inflammasome activation within the cochlea and retina contributes to murine neuroinflammation [44, 45]. These observations may help explain the predominant affection of the vestibulocochlear nerve, the optic nerve, and the trigeminal nerves in patients with NLRP3 variants in our study.

The majority of patients in our cohort had concomitant MS. This and the fact that most patients were adults are readily explained by the fact that our outpatient clinic is specialized on the treatment of adult MS patients [13, 35]. Former studies already suggested that AID and MS are more likely to co-occur than expected by chance [21, 26, 46]. Previous studies have identified the R92Q variant in the *TNFRSF1A* gene as a risk factor for MS [47]. The high prevalence of this variant in our study supports these observations. In contrast, although the Q703K NLRP3- and the E148Q MEFV variants have been described in MS patients [24, 28], they have not been confirmed as a susceptibility factor for MS so far. Interestingly, we observed both the K695R and the R408Q (in six cases as a compound heterozygous P369S-R408Q mutation) variants almost exclusively in MS patients, which warrants further investigation in larger study populations. Overall, the disease course of MS in AID patients was similar to MS patients without mutations. This suggests that the underlying genetic variants serve more as a susceptibility factor rather than being a distinct disease modifier in MS [24, 26, 28]. In addition, treatment with colchicine and anti-IL-1 therapy had no major influence on the disease course of MS in our small patient cohort.

It can be difficult to distinguish between “MS with coexisting AID” and “AID with CNS involvement” since the clinical presentation may be similar. Both disorders manifest with variable and episodic symptoms, often affect the optic nerve, and show white matter lesions on MRI as well as abnormalities in the CSF. Thus, careful evaluation and follow-up investigations are of great importance. CAPS, FMF, and TRAPS usually occur with multisystemic inflammation and therefore require medical care and treatment by a collaborative, multidisciplinary team. If at all, adult patients with a presumed diagnosis of an AID are seen by rheumatologists first. As a consequence, neurological manifestations may be overlooked, as neurological examinations as well as MRI and CSF analysis are not routinely performed in such settings.

Although we observed some differences concerning systemic symptoms, overlapping systemic manifestation makes it difficult for the clinician to distinguish between NLRP3-, MEFV-, or TNFRSF1A variants. Thus, if an AID is suspected, we propose to perform a multipaneled genetic diagnostic test as it is offered now in many laboratories. In addition, it can be challenging to differentiate AID from other rheumatological diseases. This often results in delayed diagnosis of AID, in fact, a majority of our patients had a mean diagnosis latency of 10 years.

The strength of this study is its sample size and its monocentric character, thereby circumventing the bias of multiple study sites. Obvious limitations, on the other hand, involve the retrospective character, recruitment bias (as an outpatient clinic specialized in MS), and the lack of patients with clear disease-causing mutations in the NLRP3- and TNFRSF1A gene for comparison. In addition, although the disease course of MS with and without genetic variants did not differ in our patient cohort, we cannot exclude an influence of these variants on MS development and progression. Since we did not collect the respective biomaterial from all our patients, we were unable to investigate and compare cytokine profiles and immune cell subtypes in sera and CSF in MS patients with and without NLRP3-, MEFV-, and TNFRSF1A low-penetrance variants. This is, however, of great interest since increased levels of IL-1ß have been reported in the CSF and in cerebral lesions of MS patients. Furthermore, IL-1β has recently been shown to correlate with cortical pathology load in MS at clinical onset [48, 49]. Additionally, a role of the NLRP3 inflammasome has been demonstrated in the development of experimental autoimmune encephalomyelitis (EAE) [50]. Taken together, a more comprehensive immunophenotyping in correlation to clinical phenotypes in MS patients with and without AID is warranted and may elucidate the impact of these genetic variants on MS disease course in future studies.

## Conclusions

Taken together, a broad spectrum of neurological manifestations including coexisting MS may occur in patients with low-penetrance variants of the *TNFRSF1A*-, *NLRP3*-, and *MEFV* genes and both neurologists and rheumatologists should keep this in mind. Diagnosis of AID should be considered a potential differential diagnosis in patients with recurrent episodes of systemic inflammation with CNS involvement and unexplained CN affection.

## Supplementary information

**Additional file 1: Figure S1.** Follow-up of AID patients w/o MS. Follow up time (in years) of NLRP3-, MEFV- and TNFRSF1A low penetrance variants w/o MS are depicted.

## Data Availability

All data generated or analyzed during this study are included in this published article.

## Abbreviations

AID: Autoinflammatory diseases
CAPS: Cryopyrin-associated periodic syndromes
CN: Cranial nerve affection
EDSS: Expanded Disability Status Scale
EAE: Autoimmune encephalomyelitis
FMF: Familial Mediterranean fever
IL-1β: Interleukin-1 beta
MS: Multiple sclerosis
MSSS: Multiple Sclerosis Severity Scale
PPMS: Primary progressive MS
SPMS: Secondary progressive MS
TNF: Tumor necrosis factor
TRAPS: Tumor necrosis factor receptor-associated periodic syndrome
vs.: Versus
w/o: Without
