# Supplementary material for: Neurological phenotypes in patients with NLRP3-, MEFV-, and TNFRSF1A low-penetrance variants
Source: J Neuroinflammation. 2020 Jun 20;17:196. doi: 10.1186/s12974-020-01867-5 (PMC7306142; doi:10.1186/s12974-020-01867-5)
Supplement: Supplementary file 1 — Additional file 1: Figure S1. Follow-up of AID patients w/o MS. Follow up time (in years) of NLRP3-, MEFV- and TNFRSF1A low penetrance variants w/o MS are depicted. [file 12974_2020_1867_MOESM1_ESM.pdf]

ID	FOLLOW-UP *IN YEARS*
NLRP3_AID-1	5
NLRP3_AID-2	9
NLRP3_AID-3	7
NLRP3_AID-4	6
NLRP3_AID-5	14
NLRP3_AID-6	10
NLRP3_AID-7	4
NLRP3_AID-8	8
NLRP3_AID-9	8
NLRP3_AID-10	10
NLRP3_AID-11	7
NLRP3_AID-12	7
NLRP3_AID-13	6
NLRP3_AID-14	4
NLRP3_AID-15	9
NLRP3_AID-16	6
NLRP3_TNFRSF1A-AID-17	12
MEFV_AID-1	4
MEFV_AID-2	14
MEFV_AID-3	11
MEFV_AID-4	11
MEFV_AID-5	10
MEFV_AID-6	9
MEFV_AID-7	8
MEFV_AID-8	8
MEFV_AID-9	7
MEFV_AID-10	6
MEFV_AID-11	6
MEFV_AID-12	5
MEFV_AID-13	5
MEFV_AID-14	6
MEFV_AID-15	9
MEFV_AID-16	10
MEFV _AID-17	10
MEFV _AID-18	4
MEFV _AID-19	12
TNFRSF1A_AID-1	12
TNFRSF1A _AID-2	14
TNFRSF1A _AID-3	14
TNFRSF1A _AID-4	12
TNFRSF1A _AID-5	12
TNFRSF1A _AID-6	12

TNFRSF1A _AID-7	15
